# Cognitive ability, gender, and well-being in school contexts: longitudinal evidence from Sweden

**DOI:** 10.3389/fpsyg.2024.1396682

**Published:** 2024-09-17

**Authors:** Björn Boman, Marie Wiberg

**Affiliations:** ^1^Department of Political Science, Stockholm University, Stockholm, Sweden; ^2^School of Business, Economics, and Statistics, Umeå University, Umeå, Sweden

**Keywords:** cognitive ability, well-being, intelligence, gender, longitudinal analysis

## Abstract

While well-being does generally constitute a moderate predictor of school achievement, research on the predictive validity of cognitive ability for well-being in school contexts remains scant. The current study analyzed longitudinal relations between cognitive ability measured at age 13 (Grade 6) and well-being measured at age 18 (Grade 12, valid *N* = 2,705) in a Swedish sample, using several multivariate model techniques. The results indicate that cognitive ability was not a statistically significant predictor when several predictors were entered in a multiple regression model. However, gender was a significant covariate as girls and young women have a substantially lower degree of self-reported well-being. This casts light on the limitations of cognitive ability as a construct for some non-cognitive outcomes, at least in shorter and narrower spatial–temporal contexts.

## Introduction

Cognitive ability, as measured by standardized IQ tests or similar cognitive assessments, is associated with a variety of positive outcomes such as academic achievement, future income, health, and well-being (e.g., Calvin et al., 2017). At least some of these positive patterns have been found across many national contexts such as China, France, Sweden, the UK, and the US (e.g., Boman, 2023a; Deary and Johnson, 2010; Deary et al., 2007; Guez et al., 2018; Laidra et al., 2007; Li et al., 2019). Moreover, cognitive ability is typically a stronger predictor of school results than other pivotal factors such as self-control, conscientiousness, or similar non-cognitive constructs (Boman, 2023a; Vazsonyi et al., 2022), although earlier research has found different patterns in smaller samples (e.g., Duckworth and Seligman, 2005). To some extent, individual-level associations between cognitive ability and educational achievement are congruent with national level estimates such as relations between average annual income and aggregated test scores (e.g., Boman, 2023b). Among the secondary factors for academic achievement, not only conscientiousness but also emotional stability is associated with higher grade point averages and test scores (e.g., Mammadov, 2022; Poropat, 2009). To some degree that is also the case of agreeableness and openness to experience (e.g., Andersen et al., 2020; von Stumm et al., 2011). However, the effects of agreeableness and openness disappear when cognitive ability is included in the same multivariate model (Mammadov, 2022). In other words, cognitive ability, as a stronger predictor, seems to cancel out the effects of the less pertinent personality factors. What about the relations between cognitive ability and well-being?

There are some studies which in part have focused on relations between cognitive ability and well-being (Klapp et al., 2024; Giota and Gustafsson, 2017). For example, Giota and Gustafsson (2017) found that students with strong inductive reasoning skills were less prone to school-related stress. However, the authors did not use a composite measure of cognitive ability. In the case of Klapp et al. (2024), the authors focused on cohort differences within the Swedish compulsory education system rather than developmental trajectories (e.g., Grade 6 to Grade 12 development). Moreover, their main focus was not on the relations between cognitive ability and well-being.

Bergold et al. (2015) examined differences in life satisfaction between gifted and non-gifted students in Germany. Their results indicate there were no substantial differences between the groups, although girls were less satisfied with their lives. While the study was well-crafted it had a cross-sectional design and was based on a relatively small sample (total *N* = 655). Nevertheless, the results echo those of Zeidner (2021).

Thus, when considering previous evidence, it appears likely that there is a positive relationship between cognitive ability and constructs which are associated with self-reported emotional stability and well-being. Earlier research has found such relationships in medical contexts (e.g., Leavitt et al., 2017; Sutin et al., 2021; Stephan et al., 2022). On the other hand, because emotional stability is at most a moderate predictor of academic achievement in K-12 education (Andersen et al., 2020; Mammadov, 2022; Poropat, 2009), it is also plausible that there exist a “null effect” regarding the relations between self-rated emotional stability or well-being and phenotypic (measured) intelligence, or that students, via academic achievement aspiration, lead more stressful lives (Heller-Sahlgren, 2018). A previous study by Weyns et al. (2021) indicates that high-ability students showed lower teacher conflict, higher peer acceptance, and better school well-being than average-ability students. Furthermore, the analyses demonstrated that peer acceptance consistently predicted school well-being over time, while Grade 4 school well-being impacted Grade 5 teacher conflict.

However, no mediating effects have been examined in that respect. It is possible that female students have lower degrees of self-reported well-being or neuroticism regardless of cognitive ability (e.g., Bergold et al., 2015; Giolla and Kajonius, 2018; Brännlund and Edlund, 2020). Hence, it constitutes both an important control variable and a potential mediator in this respect. In addition, the above-mentioned study focused on lower grade levels when cognitive and non-cognitive traits are less stable (Mammadov, 2022). Hence, it is important to replicate the findings with gender related mediation models as well as with large samples consisting of older students. This does also enable an examination of transition phases from compulsory school to the upper-secondary educational level.

## The current study

Overall, more research is required with regard to the specific relations between cognitive ability (or intelligence) and self-reported well-being or emotional stability, especially in school and adolescence contexts. There are two gaps in the literature that need to be considered in particular. One concerns the predictive validity of cognitive ability for well-being, while the other concerns study design. For example, cognitive ability is a stable predictor of academic achievement, health, income, and wealth (e.g., Deary and Johnson, 2010; Marks, 2022) but it is less clear if it is for well-being. Moreover, the developmental trajectories should be longer than only a few years such as when measurement points of non-cognitive abilities in Grade 6 predicting achievement in Grade 8 or Grade 9 (c.f. e.g., Boman, 2023a; Giota and Gustafsson, 2021). Bronfenbrenner (1994) stresses that individuals develop within both micro (e.g., family units) and meso contexts (e.g., schools), partially affected by exosystemic and macrosystemic factors such as national curricula, regulations, and cultural beliefs. Therefore, it might be important to focus on specific educational contexts in particular countries (Boman and Wiberg, 2024).

Moreover, time (i.e., chronosystems) is an important developmental factor for children and adolescents and therefore longitudinal relationships and multiple points of measurement are pertinent (Bronfenbrenner, 1994). Maxwell et al. (2011) accentuate that mediating processes require time to have a substantial effect. For example, even if cognitive ability, SES, and gender are measured early in life and are more or less static factors they may have direct and indirect effects through much of the adolescent years and beyond (Boman, 2023a). Furthermore, Malanchini et al. (2024) underscore that it is crucial to have a substantial temporal gap between the years of measurement as regards educational achievement research. Therefore, the current study used data points when students were 13 years old (i.e., Grade 6) to predict well-being when students were 18 and 19 years old and attending secondary school (i.e., Grade 12). This constitutes a longer gap compared to earlier research with similar aims and/or data (e.g., Boman, 2023a; Giota and Gustafsson, 2021). The analyses are based on both linear regression models, including interaction effects, and mediation models where both gender, cognitive ability/intelligence, and socioeconomic status (SES) are used as potential mediators. The study is considered exploratory.

In the current study, the following research questions were addressed:

1. What is the association between cognitive ability and well-being?
2. Does gender mediate the relationship between cognitive ability and well-being?

Moreover, the following exploratory hypotheses were suggested:

*H1*: There is a relationship between cognitive ability and perception of well-being.

*H2*: Cognitive ability as measured at an earlier time point (e.g., Grade 6) can partially predict well-being at a later time point (e.g., Grade 12).

*H3*: There is an association between gender and perception of well-being.

*H4*: There is an association between cognitive ability and perception of well-being which is mediated by gender.

## Method

### Data

The current study is based on the Evaluation Through Follow-up (ETF) database which has been conducted by Sweden Statistics since the 1960s (e.g., Härnqvist, 2000). The ETF data set includes multiple indicators of parental education (Svensson et al., 2007), migration background, grades and national test results for all subjects and sub-tests, as well as various cognitive test results, and non-cognitive indicators and attitudes toward their school situation and well-being, as well as information about special needs programs and physical health development. The information which the data builds upon was retrieved from the schools’ administration (e.g., grades and national test results) but also students, parents, and teachers filled in questionnaires, typically on several occasions in Grade 3, Grade 6, Grade 9, and Grade 12.

In the current study, background information (e.g., parental and family SES) was retrieved in Grade 3, while cognitive tests were administered in Grade 6. The ninth cohort was born in 1998 (*N* = 9,671, 48,6% girls) and measurement points were in 2011 (Grade 6), 2014 (Grade 9), and 2017 (Grade 12). The intermediate point of measurement, Grade 9, was deliberately omitted in order to focus on longer timeframes. Specifically, Grade 3 and Grade 6 variables were linked with their Grade 12 counterparts.

### Instruments

#### Well-being

Well-being, which was used as the main term in the current article despite the potential overlap with Big Five neuroticism/emotional stability, was measured through 11 items in Grade 12 at the upper-secondary level when students are 18 years old. These are partly consistent with previous research (Pollard and Lee, 2003). Overall, the items correspond mostly to the classification made by VanderWeele et al. (2020). That implies that the items cover subjective well-being with both psychological and physical features, as a form of hedonic well-being. In other words, it is the students’ perceptions of themselves in this respect that are being captured.

The items were usually phrased as in the following example: ‘Both in and outside school, during the last 6 months, have you experienced difficulty in concentrating?’. The last word was replaced with similar words or phrases such as ‘difficulty in sleeping,’ ‘suffered from headaches,’ ‘felt sad,’ ‘felt nervous,’ and so forth. These items were measured on a Likert scale where 1 = Always/Almost always, 2 = Often, 3 = Sometimes, 4 = Rarely, 5 = Never/Almost never.

Cronbach’s alpha for the 11 items was *α* = 0.910 (valid *N* = 3,652 with listwise deletion). According to Kline (2016), high alpha numbers may be present when there are many items with strong internal consistency. The authors decided to include all items which constitute the construct. This is also the study’s dependent variable.

Moreover, satisfaction with school (measured in Grade 6) was included by using a set of items that focus on how content students are with their school situation in terms of their pupils, class, teachers, school, and so forth (e.g., Andersen et al., 2020). This variable was included in one of the regression models as its correlation with emotional stability was only *r* = −0.134, and thus leading to no problems with multicollinearity (Cohen, 1988; Kline, 2016). Taken at face value, the items that represent school satisfaction seem to be conceptually related to school belongingness (e.g., Alverson, 2014; Malone et al., 2012). Theoretically and empirically, both well-being (both within and outside of school contexts) and school belongingness (i.e., level of content in the school context) demonstrate the interplay between micro and meso systems (e.g., Bronfenbrenner, 1994), specifically how school students perceive their situation in relation with various cognitive and non-cognitive factors.

#### Cognitive ability

The cognitive ability test which is a part of the EFA data is described by Svensson (1964) as having both verbal, spatial and inductive characteristics. The data set includes four different cognitive ability measures which to different extents are linked to fluid and crystallized dimensions of cognitive abilities: antonyms, synonyms, reversed number series, and metal folding. Metal folding is the item which is the most reminiscent of Raven’s progressive matrices while the others are mainly associated with verbal abilities. All items seem to represent sub-dimensions of the general factor of intelligence, *g* (Gustafsson, 1984; Catell, 1987; Giota et al., 2008). The number of correct answers on these four sub-tests were used as a continuous measure of the cognitive ability levels among the Grade 6 schoolchildren. Cronbach’s alpha for the 1998 cohort was acceptable (*α* = 0.732, valid cases *N* = 7,682).

### Covariates

In accordance with, for instance, Boman (2023a) and Mammadov (2022), the current study included the most common and relevant covariates as control variables: socioeconomic status, SES (measured at *T1*, Grade 3), gender (measured at *T1*, Grade 3), migration background (measured at *T1*, Grade 3), and achievement-oriented non-cognitive factors beyond well-being (which are defined synonymously in the current research context), measured at *T2* (Grade 6).

### SES

In accordance with the suggestions conveyed by Svensson et al. (2007), the current study focused on a SES measure that covers parental education in a way that reflects parents that are predominantly born in the 1970s. It consists of 11 categories where 1 represents pre-secondary education, and a doctoral degree represents 11. The intermediate levels of education consist of various degrees of secondary and tertiary education, as well as licentiate degree (=10) which means a half doctoral degree (2 years post-master’s level). This information was then captured by a four point variable that measured the highest educational level by both the biological parents and the parents living with the child. While complete SES consists of a composite of parental education, parental income, and occupational position (Sirin, 2005; Sackett et al., 2009), many researchers consider a single SES indicator to be sufficient (e.g., Falk et al., 2021; Wiberg and Rolfsman, 2023).

### Migration background

A dichotomous migration background variable, which consists of two options, Swedish = 0, Immigrant = 1, was included as a control variable (e.g., Boman, 2023a).

### Gender

Gender was included as a binary variable where females take the value 1 and males the value 0. Although there is a non-binary response option, this was not included in the current study as the study focuses more on the biological sex differences. According to earlier research on gender differences in personality constructs such as the five-factor model (e.g., Giolla and Kajonius, 2018; Weisberg et al., 2011), it is expected that females may have substantially higher levels of self-rated neuroticism or similar constructs. Hence, it might be a strong predictor or mediator in relation to the current research topic (e.g., Schmitt, 2007). Because of the coding patterns, a negative relationship between females (=1) and well-being (lower numbers = higher well-being) was expected.

### Non-cognitive abilities

Non-cognitive abilities that are pertinent for school results and well-being were included. These items constitute indicators of confidence in one’s abilities within the frames of the school context. These are self-rated (Giota et al., 2008; see also Andersen et al., 2020). There were in total five non-cognitive items within the EFA questionnaire that were related (e.g., Rammstedt and John, 2007). For example:

- “How true are the following statements: I can normally manage to do the tasks that I am given.”
- “How true are the following statements: I can normally answer the questions that I am given correctly.”

Cronbach’s alpha was acceptable (*α* = 0.702, valid cases *N* = 7,839) and therefore all five items made up a composite variable.

### Analytical procedures

The study is based on multivariate techniques such as confirmatory factor analysis, linear regression analyses, and mediation models which measure both direct and indirect relations (e.g., Kline, 2016). The CFA aimed to establish if a well-being construct exists and was conducted in Mplus 8.7 (Muthén and Muthén, 2020). Specifically, the 11 well-being items were hypothesized to constitute a single latent factor. Four fit indices were examined: the incremental goodness of fit index CFI (Comparative Fit Index, cut-off value >0.95), which compares the fitted model with an ill-fitting baseline model, as well as the RMSEA (Root Mean Square Error of Approximation, cut-off value <0.08), the SRMR (Standardized Root Mean Square Residual, cut-off value 0.08), and the Chi-square test. Albeit a statistically significant Chi-square value (e.g., 0.000) represents a poor model fit, it is often expected in large sample studies (Hu and Bentler, 1999; Kline, 2016). The Maximum Likelihood Robust (MLR) imputation was used.

The main multiple regression models (Model 1–2), which were conducted in SPSS 29, aimed to demonstrate the predictive value of cognitive ability (Grade 6, *T2*) on well-being (Grade 12, *T3*). Except for the control variables stated above, the model also controlled the level of content in schools that students reported in Grade 6. The variables that were included in the regression models were analyzed through the multiple imputation function for missing values, which are often quite large in longitudinal data sets. However, the missing values had a random constitution and did not lead to biased regression estimates.

**Figure 1:**
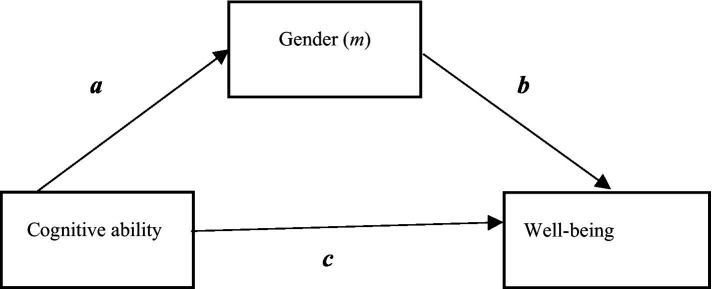
Assumed relationships. The model shows the assumed relationships of a main mediation model where gender mediates the relationship between cognitive ability and well-being and all paths are statistically significant.

**Figure 2:**
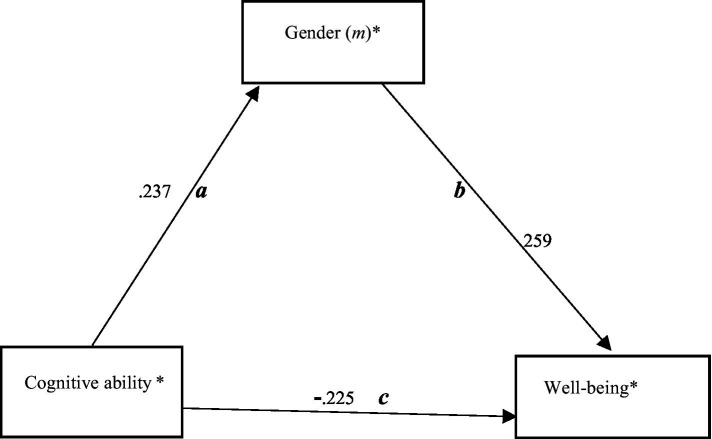
Fitted meditation relationships. The model shows the fitted relationships of a mediation model where cognitive ability is an independent variable, gender is the mediator, and well-being the dependent variable.

**Figure 3:**
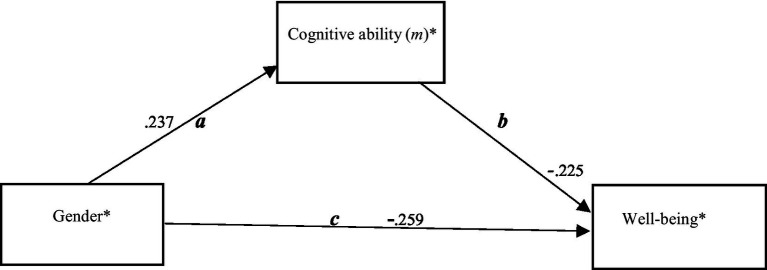
Fitted mediation relationships. The model shows the fitted relationships of a mediation model where gender is an independent variable, cognitive ability is the mediator, and well-being the dependent variable.

Additional mediation models aimed to examine indirect effects of gender in relation to cognitive ability which was suggested to be an important predictor or mediator. These are part of an exploratory framework, although earlier research in the Swedish context indicates that gender might be a mediator between intelligence or academic achievement and well-being (Giolla and Kajonius, 2018; Brännlund and Edlund, 2020). These models were conducted in Mplus 8.7. According to Zhao et al. (2010), the basic assumption with regard to mediation models is that the direct effects of the *x* variable (i.e., the independent variable) must be statistically significant and that is also the case with the mediator (*m*). Then the direct and indirect effects of the predictors estimate the model fit (Zhao et al., 2010). Overall, we examined eight different versions of the model shown in Figure 1 but only two models of theoretical interest are displayed in the manuscript (Figures 2, 3). The longitudinal mediation model had the following equational constitution:


$$M_{st}+1=M_{st}{+}_{a}X_{st}+bX_{st}+e_{st}+1\text{,}$$

$$\mathrm{Wellbeing}12_{s}+1=Y_{st+1}+bM_{\mathrm{it}}+cX_{\mathrm{it}}+e_{st}+1\text{,}$$

where *M_st_* + 1 is the score for student *s* on variable *M* at time *t* + 1, *M* it is the score for student *s* on variable *M* at time point *t*, *aX_st_* is the score for individual *s* on variable *aX* at time *t*, *bX_st_* is the score for individual *s* on variable *bX* at time *t*, and *eM_st + 1_* is an error term reflecting other influences on *M*. *Wellbeing12_s_* is the Wellbeing score, whereas *bM_it_* is the mediating factor, *cX_it_* the direct effect of the independent variable *x* on *y*, while *e_st + 1_* is the error term for the second part of the equation.

## Results

The results from the CFA (*X^2^* = 2986.36, RMSEA = 0.139, CFI = 0.946, SRMR =0.048) showed a relatively good model fit for the latent well-being variable, which indicates that it is a unified construct. However, as has been noted by Lai and Green (2016) our fit indices are slightly below some of the conventional thresholds.

The results of the two regression models (Tables 1, 2) showed that only gender was statistically significant with a rather large effect size (e.g., *β* = −0.369). That means that 18- or 19-year-old women experience lower levels of physical and psychological well-being compared to young men of a similar age. Cognitive ability was not statistically significant in any of the two models. This does also suggest that young women have lower levels of self-reported well-being regardless of their cognitive ability levels. No interaction effects (gender*cognitive ability) were found in consecutive analyses.

**Table 1:** Regression results (model 1).

Variable	*B*	*β*	Standard error	*P*-value
(Constant)	37.245		1.126	<0.001
Cognitive ability	−0.002	−0.004	0.010	>0.10
Gender	−0.135	−0.365	0.006	<0.001
Migration background	−1.730	−0.056	0.553	<0.005
SES	0.616	0.034	0.335	<0.010
Non-cognitive	0.784	0.092	0.162	<0.001

The table shows the regression results where cognitive ability is the main independent variable. Only the constant and gender are statistically significant at the 1% level. The dependent variable is well-being measured at Grade 12 (T3). *R*^2^: 0.150. *N* = 2791.

**Table 2:** Regression results (model 2).

Variable	*B*	*β*	Standard error	*P*-value
(Constant)	41.451		1.259	<0.001
Cognitive ability	0.002	0.005	0.010	>0.10
Gender	−0.137	−0.369	0.007	<0.001
Migration background	−1.820	−0.059	0.551	<0.001
SES	0.001	0.035	0.337	>0.10
Non-cognitive	0.474	0.055	0.173	<0.005
School satisfaction	−0.328	−0.123	0.050	<0.001

The table shows the regression results where cognitive ability is the main independent variable. Only the constant, the level of content in school (reverse coded), and gender are statistically significant at the 1% level. The dependent variable is well-being measured at Grade 12 (T3). *R*^2^: 0.168. *N* = 2,705.

The second regression model (Table 2), which included a variable that captured previous level of school satisfaction (*T2*, Grade 6), showed similar relationships. However, when this model added a partly similar construct (i.e., content in the school), the non-cognitive factor had a higher *p-value* (<0.005 compared to <0.001). This is likely because these constructs, in part, overlap. All in all, there is virtually no substantial relation between cognitive ability and well-being as far as the regression analyses go. However, the bivariate correlation between cognitive ability and well-being is positive, albeit small in size.

The main mediation models (Figures 2, 3) show that there is a partial mediation (Maxwell et al., 2011) between cognitive ability, gender, and self-reported well-being, as well as between gender, cognitive ability, and well-being. Female students have higher cognitive ability and lower self-reported well-being. Both gender and cognitive ability have the function as mediators.

## Discussion

### Summary of the results

The current study examined the relationships between cognitive ability and self-reported well-being in a school context by taking advantage of a relatively large cohort of students in Sweden from the education through follow-up (EFA) database. The findings indicate that the association between cognitive ability and well-being is negligible, which suggests that students’ cognitive ability might be independent of their well-being, or that (measured) cognitive ability levels are distributed equally across groups with relatively higher or lower levels of self-perceived well-being. Instead, gender differences explain the largest amount of variance regarding well-being, partly in line with another register-based study in Sweden (Brännlund and Edlund, 2020). Therefore, the third hypothesis was confirmed.

Overall, the findings indicate that there is not a meaningful relationship between cognitive ability and well-being, at least in this particular educational context (i.e., Sweden). While emotional stability has a small to moderate relation with school results (e.g., Mammadov, 2022), it seems that cognitive ability is not substantially related to subjective and hedonic well-being, which is a similar but not identical construct. Thus, the two exploratory hypotheses were at most only partially confirmed.

Instead, it appears as if the control and mediating variable, gender, has the largest effect on well-being at age 18 or 19 (Grade 12), even when controlling for earlier level of content or satisfaction in the school context, SES, and other covariates. This particular finding is consistent with Giolla and Kajonius (2018) and Brännlund and Edlund (2020), who underline that gender differences in personality, well-being or health are somewhat larger in countries such as Sweden which are characterized by a higher level of gender equality. Other covariates such as SES, non-cognitive factors, or migration background did not contribute substantially to the multivariate models. Only gender increased the *R^2^* values in the regression models considerably.

The pertinence of gender might be related to the fact that girls outperform boys in the Swedish school context and other national contexts, at least until a typical post-tertiary education age has been surpassed. These includes broader cognitive skills that tap into cognitive ability (Borgonovi et al., 2021). Hence, girls work hard for their school grades at the upper-secondary level, and in tandem with stress, hormonal and temperamental fluctuations throughout the menstrual cycle (e.g., Peters et al., 2020) this may lead to recurring experiences of emotional instability and physical problems (e.g., stomachache and headaches). Moreover, girls have higher cognitive ability at least until age 13, and perhaps even until later years (e.g., Borgonovi et al., 2021). Hence, if males cognitively catch up with females at an adult age the cognitive ability-well-being nexus might have a different constitution.

### Implications, limitations, and directions for future research

The study contributes to the ongoing scholarly discussion on cognitive ability and its relationship with other important factors or outcomes such as academic achievement, personality, gender, and well-being in school contexts (e.g., Bergold et al., 2015; Giota and Gustafsson, 2021; Weyns et al., 2021; Marks, 2022). While the results might be restricted to the Swedish context (e.g., Boman, 2023a; Giota and Gustafsson, 2021) the study nonetheless adds to the international corpus in this regard.

The current study, which was based on one Swedish cohort of school students, has several limitations. As is the case with many longitudinal data sources, more values are missing at the latest points of measurement which in this case are in Grade 12 (*T3*) compared to Grade 3 (*T1*) and Grade 6 (*T2*). Yet, the sample size is relatively large (*N* = 2,705) and thus the authors are rather confident in its reliability. However, it remains unknown if these results could be transferred to other developmental or national contexts. Moreover, compared to several medical studies the current study has a limited timeframe (6 years). Nonetheless, regarding educational contexts and transition phases this constitutes a substantial and important timeframe (e.g., see Malanchini et al., 2024).

There are also other ways to measure well-being, particularly emotional stability, such as through the neuroticism items that are part of the Five-factor Model (e.g., Rammstedt and John, 2007). Hence, future research may combine and compare well-being constructs with Five-factor Model constructs in this regard.

Furthermore, there are other limitations. For example, we did not measure relations between well-being, cognitive ability, and academic achievement as these were not part of our exploratory study. Overall, we consider these relations to be well-established and therefore of less scientific novelty. However, during exploration processes of the data set, the authors found substantial relations between cognitive ability (measured in Grade 6) and grade point average in Grade 12. Moreover, some variables were only measured in Grade 6 (e.g., school content). This is because we deliberately omitted intermediate years of measurement (Grade 9) to investigate longer developmental trajectories. Some variables do also have only one point of measurement.

In addition, many of the covariates are, to a degree, intercorrelated and might therefore overlap. For example, self-rated abilities (i.e., non-cognitive abilities) tap into cognitive abilities and higher SES are associated with higher cognitive ability. However, these intercorrelations are small (*r* = 0.30<) with regard to the current data set and in general (Marks, 2022; Marks and O’Connell, 2021). That is also why the mediation models may omit covariates. Broader path models are based on regression coefficients (Kline, 2016). Hence, our linear regression models complement the mediation models. Moreover, we had little theoretical interest in other covariates than cognitive ability, well-being and gender.

Nevertheless, our study contributes with knowledge on the under-researched topic of the relations between cognitive ability, well-being and gender, using high-quality longitudinal data. We find that gender partially mediates the relations between cognitive ability and well-being, and that gender is a significant predictor of well-being as female students have lower levels of self-reported well-being. Moreover, we less expectedly found that cognitive ability partly mediates the association between gender and well-being.

## Data Availability

The original contributions presented in the study are included in the article/supplementary materials, further inquiries can be directed to the corresponding author.
